# Lipopolysaccharide impairs amyloid beta efflux from brain: altered vascular sequestration, cerebrospinal fluid reabsorption, peripheral clearance and transporter function at the blood–brain barrier

**DOI:** 10.1186/1742-2094-9-150

**Published:** 2012-06-29

**Authors:** Michelle A Erickson, Pehr E Hartvigson, Yoichi Morofuji, Joshua B Owen, D Allan Butterfield, William A Banks

**Affiliations:** 1 Department of Pharmacology and Physiology, Saint Louis University, 1402 South Grand Blvd, St. Louis, MO, 63104, USA; 2 GRECC, Veterans Affairs Puget Sound Health Care System, 1600 S. Columbian Way, Seattle, WA, 98108, USA; 3 Division of Gerontology and Geriatric Medicine, Department of Internal Medicine, University of Washington School of Medicine, Bldg. 1, Rm. 810A, 1660 Columbian Way, Seattle, WA, 98108, USA; 4 Department of Chemistry, Center of Membrane Sciences and Sander-Brown Center on Aging, University of Kentucky, 249 Chemistry-Physics Building, Lexington, KY, 40506, USA

**Keywords:** Alzheimer’s disease, amyloid beta, blood–brain barrier, inflammation, lipopolysaccharide, LRP1, Pgp, ABCB1, MDR1, cerebrospinal fluid

## Abstract

**Background:**

Defects in the low density lipoprotein receptor-related protein-1 (LRP-1) and p-glycoprotein (Pgp) clearance of amyloid beta (Aβ) from brain are thought to contribute to Alzheimer’s disease (AD). We have recently shown that induction of systemic inflammation by lipopolysaccharide (LPS) results in impaired efflux of Aβ from the brain. The same treatment also impairs Pgp function. Here, our aim is to determine which physiological routes of Aβ clearance are affected following systemic inflammation, including those relying on LRP-1 and Pgp function at the blood–brain barrier.

**Methods:**

CD-1 mice aged between 6 and 8 weeks were treated with 3 intraperitoneal injections of 3 mg/kg LPS at 0, 6, and 24 hours and studied at 28 hours. ^125^I-Aβ_1-42_ or ^125^I-alpha-2-macroglobulin injected into the lateral ventricle of the brain (intracerebroventricular (ICV)) or into the jugular vein (intravenous (IV)) was used to quantify LRP-1-dependent partitioning between the brain vasculature and parenchyma and peripheral clearance, respectively. Disappearance of ICV-injected ^14^ C-inulin from brain was measured to quantify bulk flow of cerebrospinal fluid (CSF). Brain microvascular protein expression of LRP-1 and Pgp was measured by immunoblotting. Endothelial cell localization of LRP-1 was measured by immunofluorescence microscopy. Oxidative modifications to LRP-1 at the brain microvasculature were measured by immunoprecipitation of LRP-1 followed by immunoblotting for 4-hydroxynonenal and 3-nitrotyrosine.

**Results:**

We found that LPS: caused an LRP-1-dependent redistribution of ICV-injected Aβ from brain parenchyma to brain vasculature and decreased entry into blood; impaired peripheral clearance of IV-injected Aβ; inhibited reabsorption of CSF; did not significantly alter brain microvascular protein levels of LRP-1 or Pgp, or oxidative modifications to LRP-1; and downregulated LRP-1 protein levels and caused LRP-1 mislocalization in cultured brain endothelial cells.

**Conclusions:**

These results suggest that LRP-1 undergoes complex functional regulation following systemic inflammation which may depend on cell type, subcellular location, and post-translational modifications. Our findings that systemic inflammation causes deficits in both Aβ transport and bulk flow like those observed in AD indicate that inflammation could induce and promote the disease.

## Background

Alzheimer’s disease (AD) is the most common form of senile dementia [1] and according to the amyloid cascade hypothesis results from the accumulation of amyloid beta (Aβ) in the brain [2]. The neurovascular hypothesis of Zlokovic states that a critical pathological event driving Aβ accumulation in brain is the reduced clearance of Aβ from brain across the blood–brain barrier (BBB) [3]. A large body of work from multiple groups has shown that the low-density lipoprotein receptor-related protein-1 (LRP-1) transports Aβ across the BBB in the brain-to-blood direction, and becomes deficient in AD [4-8]. Evidence for LRP-1 deficiency includes the correlation of LRP-1 levels with impairment of Aβ efflux in rodent models of AD [4], LRP-1 downregulation in brain microvasculature of patients with AD [8], and oxidative modification of LRP-1 in AD hippocampus [9]. Growing evidence also supports a role of a second efflux transporter, p-glycoprotein (Pgp), in Aβ efflux across the BBB [10]. Similar to LRP-1, results suggest that Pgp dysfunction also occurs in AD [11,12]. Despite data supporting a deficiency in Aβ transport in AD, little is known about the mechanisms that could initiate or sustain these transport deficiencies in disease progression.

Two other processes which are thought to contribute to the level of Aβ in brain are clearance through bulk flow of cerebrospinal fluid (CSF), and clearance of Aβ from the periphery. Bulk flow denotes the reabsorption of CSF into peripheral compartments, including the systemic circulation [13]. Inhibition of CSF turnover occurs in AD, and is thought to contribute to buildup of potential toxins, including Aβ, in the AD brain [14]. Furthermore, reduction of Aβ in brain restores bulk flow in an AD mouse model [15]. Therefore, deficiency of CSF turnover reflects an important pathophysiological consideration in AD. Multiple groups have shown that peripheral clearance of Aβ is important in regulating Aβ levels in brain and may decrease in AD. This clearance occurs primarily through the liver and kidney [16], and LRP-1 has been identified as a primary transporter for uptake of Aβ by liver [17]. Two potential mechanisms could explain how decreased clearance of Aβ from blood contributes to accumulation in brain. First, the receptor for advanced glycation endproducts (RAGE) has been identified as an influx transporter for Aβ [18]. Therefore, decreases in peripheral clearance of Aβ would promote entry into the brain. Second, decreased Aβ clearance from the periphery is associated with impaired efflux [19]. The mechanism governing this phenomenon is presently unclear, but could be attributed to concentration gradients or endothelial dysfunction due to RAGE activation [20]. Understanding the mechanisms that contribute to impairment of BBB efflux, CSF bulk flow, and reduced peripheral clearance of Aβ in AD may provide clues for the important early stages of AD pathogenesis.

Inflammation and oxidative stress in the brain are concurrent with AD and roles for each in the pathogenesis of AD have been proposed. Aβ causes inflammation in the brain through Toll-like receptor and complement activation [21-23]. Elevated levels of proinflammatory cytokines and acute phase proteins are localized around Aβ plaques in AD [21], suggesting that the AD brain is in a chronic proinflammatory state. Oligomeric Aβ_1-42_ can also cause oxidative stress by integrating into membranes and catalytically generating the lipid peroxidation product, 4-hydroxynonenal (HNE) [24], and through activation of the ROS-generating enzyme NADPH oxidase in microglia [25]. In addition to causal roles of Aβ initiating neuroinflammation and oxidative stress, inflammation and/or oxidative stress can themselves cause Aβ accumulation in the brain. The amyloid precursor protein, from which Aβ is cleaved, is transcriptionally regulated similarly to heat shock proteins and is responsive to the proinflammatory cytokine IL-1 [26]. Others have shown that lipopolysaccharide (LPS)-induced inflammation increases Aβ accumulation and deposition in brain [27,28]. Oxidative stress upregulates proteins involved in Aβ production, such as presenilin 1 [29]. Because Aβ is present in brain under physiological conditions and upregulated by stressors, some have postulated that Aβ plays important roles in the stress response. Modest, transient upregulation of Aβ in the brain may serve as an antioxidant defense [30] and promote clearance of damaged cells in the brain by microglia [31]. Under severe or chronic conditions of cellular stress, it is therefore feasible that Aβ accumulation could transition to pathological levels, resulting in formation of toxic oligomers that drive the AD process. This warrants further investigation into mechanisms by which inflammation and oxidative stress contribute to BBB efflux of Aβ.

Evidence supports that induction of systemic inflammation by the proinflammatory molecule LPS alters both LRP-1 and Pgp at the BBB. Our group has previously reported that peripheral administration of LPS inhibits Aβ efflux transport out of the brain [32]. LPS also is known to increase LRP-1 proteolytic processing in macrophages and neurons [33,34]. Because increased oxidative stress occurs in brains with systemic inflammation [35] and increased oxidative modifications on LRP-1 are found in AD [9], it is also possible that oxidative modifications on LRP-1 contribute to its dysfunction following LPS. Many groups have observed decreased functional Pgp in inflammatory models [36-38], including the regimen shown to impair Aβ efflux [7]. Because LRP-1 and Pgp are located at the abluminal and luminal membranes of the brain endothelial cell respectively, it has been proposed that LRP-1 facilitates the initial uptake of Aβ from the brain interstitial fluid, followed by Pgp pumping Aβ out of the endothelial cell into the blood [11]. Pgp may also regulate Aβ levels in brain by restricting the entry of circulating Aβ [39].

In this study our goal was to investigate mechanisms by which LPS alters Aβ transport out of the brain. To do this, we first measured LPS-induced changes in partitioning between the neurovasculature and parenchyma of ^125^I-labeled murine Aβ_1-42_ or the LRP-1 ligand alpha-2-macroglobulin (a2M) injected in the lateral ventricle of the brain. We then measured effects of LPS on peripheral clearance of Aβ, effects on CSF bulk flow, and changes in microvascular LRP-1 and Pgp. Our findings highlight that multiple routes of Aβ clearance are impaired by LPS, and therefore may have a synergistic effect on Aβ accumulation in brain.

## Methods

### Animal use and treatment regimens

All animal protocols were performed in an Association for Assessment and Accreditation of Laboratory Animal Care accredited facility and approved by the animal committee of the VA and St Louis University Medical Centers. Male CD-1 mice were purchased from Charles River and kept on a 12/12 hour light/dark cycle with food and water freely available. Mice at 6–8 weeks of age were treated with 3 intraperitoneal (IP) injections of 3 mg/kg LPS from *Salmonella typhimurium* (Sigma, St. Louis, MO, USA) dissolved in sterile normal saline over a 24-hour period as previously described [32]. Briefly, the first injection was given in the morning, and the second and third injections were given at 6 and 24 hours following the first injection, respectively. All mice were studied at 28 hours following the first injection. Mice given this injection regimen displayed overt sickness behavior and weight loss. No mice died as a result of this treatment regimen. A total of 225 mice were used in this study: 90 were used for detection of oxidative modifications to LRP-1 and Pgp measurement, 30 for LRP-1 measurement, 44 for measurement of Aβ and a2M vascular sequestration, 20 for CSF bulk flow measurement, 21 for measurement of peripheral Aβ clearance, and 20 for primary endothelial cell culture.

### Iodination of Aβ, a2M, and albumin

Murine Aβ_1-42_ was purchased from Bachem (Torrance, CA, USA) and bovine serum albumin (BSA) and human a2M from Sigma (St. Louis, MO, USA). Lyophilized Aβ was resuspended at a concentration of 1 mg/ml in 0.1 M ammonium hydroxide to prevent aggregation, aliquoted, and stored frozen at −80 °C for up to 3 months. Lyophilized a2M was resuspended in water at a 1 mg/ml concentration and stored at −20 °C. Activation of a2M was done by incubating in a final concentration of 0.2 M methylamine overnight at room temperature as described previously [40]. Using the chloramine-T method [41], 5 μg of Aβ, albumin, or a2M was labeled with 0.5 mCi ^125^I or ^131^I (Perkin Elmer, Waltham, MA, USA), and separated from free ^125^I on a Sephadex G-10 column (Sigma, St. Louis, MO, USA) to yield radioactively labeled Aβ (I-Aβ), albumin (I-albumin), or a2M (I-a2M). To assess stability of I-Aβ and I-albumin, an aliquot of the labeled peptide fraction was precipitated in 15% trichloroacetic acid. All iodinated proteins consistently showed greater than 95% activity in the precipitate, and I-Aβ and I-a2M was always used within 24 hours of radioactive labeling. We have found that this method of Aβ labeling shows specificity for LRP-1-dependent BBB efflux from brain [7].

### Measurement of inulin efflux

Inulin is not transported across the BBB and lacks binding sites in brain tissue [4]. Therefore, any efflux of inulin from brain would represent a bulk flow route. To measure inulin efflux, ^14^ C-inulin (Perkin Elmer, Waltham, MA, USA) was diluted to a concentration of 1 × 10^6^ CPM/μl in BSA/lactated Ringer’s solution; saline or LPS-treated mice were anesthetized with 40% urethane, and 1 μl ^14^ C-inulin was injected into the lateral ventricle of the brain (intracerebroventricular (ICV)) by reflecting the scalp and drilling a hole 1 mm lateral and 0.5 mm posterior to the bregma, followed by injection at a depth of 2.5 mm using a 26 g Hamilton syringe. Venous blood and brains were collected 10 minutes post-injection (t10). To account for central nervous system (CNS).distribution of ^14^ C-inulin, an identical treatment group was overdosed with urethane, and ^14^ C-inulin was injected ICV 10 minutes post-mortem (t0). Only brains were collected for this group. For quenching normalization of brains, 1 μl of injectate (injection check) was added in triplicate to a matrix of solubilized brain in liquid scintillation cocktail. Injection checks for serum were in liquid scintillation counter (LSC) cocktail. Radioactivity in solubilized brain and 50 μl of serum was measured using a Packard Tri-carb LSC. Brain efflux was calculated by first determining the percent of injected material remaining in brain in t10 and t0 groups:

(1) $$\%Inj/brain=100\left(CPM\;in\;brain/CPM\;injection\;check\right)$$

Delta values were calculated by subtracting individual values of %Inj/brain for each t10 mouse from the average %Inj/brain of each t0 group:

(2) $$Delta\%Inj/brain=\left(Average\%Inj/brain\;t0\right)-\left(\%Inj/brain\;t10\right)$$

Appearance of ICV-injected material in serum was calculated by determining the percent of injected material per microliter:

(3) $$\%Inj/\mu l=100\left(CPM\;in\;serum/CPM\;injection\;check\right)/50\mu l$$

### Measurement of vascular sequestration of Aβ and a2M

Vascular sequestration of ICV-injected I-Aβ or I-a2M was measured using a modified version of the capillary depletion method [42]. I-Aβ or I-a2M was diluted in BSA/lactated Ringer’s solution to a concentration of 2 × 10^5^ CPM/μl. As described under ‘Measurement of inulin efflux’, 1 μl I-Aβ or I-a2M was injected in the lateral ventricle of the brain. Blood from the jugular vein and brains were collected at 10 minutes post-injection, and brains were immediately put in ice-cold vascular depletion buffer (10 mM HEPES, 141 mM NaCl, 4 mM KCl, 2.8 mM CaCl, 1 mM MgSO_4_, 1 mM NaH_2_PO_4_, 10 mM D-glucose), and stored on ice until processing. Blood was allowed to clot at room temperature, and then centrifuged at 5,000 g to separate the serum from blood cells. Brains were homogenized with 6–8 passes of a Teflon pestle, and homogenates diluted in an equal volume of 40% ice-cold dextran (Sigma, St. Louis, MO, USA). Homogenates were centrifuged for 20 minutes at 3,500 g at 4 °C, and the parenchymal layer and dextran interface were removed and transferred to a separate tube. The remaining vascular pellet, as well as the dextran/parenchymal layers, were then counted separately in a gamma counter. Data for activity present in the vascular or parenchymal fractions were expressed as % total CPM:

(4) $$\%Total\;CPM=CPM\;in\;parenchymal\;or\;vascular\;fraction/\left(CPM\;in\;vascular\;fraction+CPM\;in\;parenchymal\;fraction\right)*100$$

### Measurement of Aβ uptake by liver and kidney, and clearance in serum

In BSA/lactated Ringer’s solution 3 × 10^5^ CPM of ^131^I-Aβ and ^125^I-albumin were prepared and injected together into the jugular vein of mice treated with LPS or saline. The liver, the left kidney, and blood from the carotid artery were collected at 1, 2, 5, 10, and 20 minutes. Blood was allowed to clot, spun at 5,000 g to separate serum from blood cells, and 50 μl serum was counted along with harvested tissues from liver and kidney in a gamma counter. The rate of I-Aβ tissue uptake was determined using multiple-time regression analysis [43]. For this analysis, experimental clock time was re-expressed as exposure time to correct for clearance of I-Aβ from the blood. Exposure time was calculated from the formula:

(5) $$Exposuretime=\left(\int_{0}^{t}\;Cpt\left(t\right)dt\right)/Cpt$$

where t equals experimental clock time, Cp represents the level of radioactivity in the serum over time and Cpt is the level of radioactivity in the serum at time t. Tissue/serum ratios were then calculated from the following formula:

(6) $$Tissue/serum\;ratio=\left(serum\;volume\right)\left(tissue\;CPM\right)/\left(tissue\;weight\right)\left(serum\;CPM\right)$$

To correct for alterations in vascular space and/or vascular permeability which occur with LPS administration, tissue/serum ratios for I-albumin were subtracted from those for I-Aβ. The corrected tissue/serum ratios were plotted against exposure time calculated for Aβ, and the unidirectional influx constant determined from the slope of the linear portion of the curve.

Serum clearance was calculated by plotting the log serum CPM/50 μl versus experimental clock time. The slope of this line is proportional to half-life by the equation:

(7) $$Half-life\;in\;serum=\log\left(2\right)/-slope$$

### Microvessel isolation

Isolation of brain microvessels from mice treated with saline or LPS was performed according to a modified protocol [44]. Briefly, three or ten brains (for western blotting and immunoprecipitation, respectively) were pooled per treatment group and homogenized in ice-cold DMEM + 0.5% PMSF with 6–8 passes of a Teflon pestle, followed by filtration once through a 300 μm nylon mesh, and twice through two 100 μm nylon mesh filters. Filtrates were then mixed with an equal volume of cold 40% dextran dissolved in DMEM, and centrifuged at 3,500 g for 30 minutes at 4 °C. The upper parenchymal layer was removed and washed once with ice-cold PBS + protease inhibitor cocktail (Sigma, St. Louis, MO, USA), and stored at −80 °C prior to protein extraction. The dextran gradient was discarded, and the microvessel pellet resuspended in DMEM. Suspended vessels were then poured onto a 25 μm mesh and washed extensively with DMEM to remove cellular debris. Washed vessels were then removed from the mesh, checked for purity by light microscopy, and washed once with PBS + protease inhibitor cocktail. The washed microvascular pellet was then stored at −80 °C prior to protein extraction. The typical microvascular protein yield for this procedure is approximately 10 μg per mouse.

### Culture of primary human, mouse, and immortalized rat brain microvascular endothelial cells and treatment with LPS

Primary human brain microvascular endothelial cells (HBECs) were purchased from Cell Systems (Kirkland, WA, USA) and cultured according to the company’s instructions. Cells at passage 6 were used in this study. Immortalized rat brain endothelial cells (RBE4), a gift from Dr Pierre Couraud, were seeded on rat tail collagen in a type 1 coated tissue culture plate (TPP, Trasadingen, Switzerland and maintained in Ham's F10/α medium 1:1 (Gibco, Invitrogen, St. Louis, MO, USA), 10% fetal bovine serum, 1 ng/ml basic fibroblast growth factor (Sigma, St. Louis, MO, USA), 300 μg/ml geneticin (Gibco, St. Louis, MO, USA), and 50 μg/ml gentamicin (Sigma, St. Louis, MO, USA). All cells were maintained at 37 °C in a humidified atmosphere of 5% CO_2_ and 95% air. Culture medium was changed twice a week, and endothelial cells at passages 7 were used in this study. Primary cultures of mouse brain capillary endothelial cells (MBECs) were isolated from 8-week-old CD1 mice according to published protocols [45] with modifications. MBECs were seeded on dishes (flasks, plates) coated with collagen type IV and fibronectin (both 0.1 mg/ml). MBEC cultures were maintained in DMEM/F12 supplemented with 10% plasma-derived serum (PDS, Animal Technologies, Inc., Tyler, TX, USA), 1% GlutaMAX supplement (Gibco, St. Louis, MO, USA), basic fibroblast growth factor (bFGF, Roche Applied Sciences, Indianapolis, IN, USA, 1 ng/ml), heparin (100 μg/ml), insulin (5 μg/ml), transferrin (5 μg/ml), sodium selenite (5 ng/ml) (insulin-transferrin-sodium selenite media supplement), and gentamicin (50 μg/ml) at 37 °C with a humidified atmosphere of 5% CO_2_/95% air; pericytes were eliminated from the culture by including puromycin (4 μg/ml) [46] in this medium (MBEC medium I). Red blood cells, cell debris, and nonadherent cells were removed 24 hours after plating by washing with medium. On the third day, the cells received a new medium which contained all components of MBEC medium I except puromycin (MBEC medium II). When the cultures reached 80% confluency (fifth day *in vitro*), the purified endothelial cells were passaged by brief treatment with 0.25% Trypsin-EDTA (Gibco, St. Louis, MO, USA) solution, and used to construct *in vitro* BBB models on transwell inserts (Corning Inc., Corning, NY, USA). All cells were treated with 0.1 mg/ml LPS dissolved in culture medium for 4 hours (HBEC) or 24 hours (MBEC and RBE4). Fresh culture medium was used as a control. Cells were then extracted for protein or fixed for immunostaining.

### Protein extraction and immunoprecipitation of LRP-1

Protein from washed cells, isolated brain microvessels, and vascular-depleted brain parenchyma were extracted in ice-cold lysis buffer (PBS plus 1% NP-40, 1 mM PMSF, and protease inhibitor cocktail) by scraping (cells) or homogenization (tissues) followed by shaking vigorously for 30 minutes at 4 °C. Extracts were then centrifuged at 20,000 g for 10 minutes at 4 °C, and supernatants were used for protein analysis. Protein was quantified in all extracts by bicinchoninic acid assay (Thermo Scientific, Rockford, IL, USA). Immunoprecipitation of LRP-1 from brain microvessel extracts was performed using a modified protocol which has been described previously [9]. Briefly, 75 μg of microvessel extract was diluted in 500 μl IP buffer (0.05% NP-40 plus protease inhibitor cocktail in PBS), and precleared by incubating with 50 μl washed protein A/G sepharose beads (Calbiochem, Billerica, MA, USA) for 90 minutes at 4 °C. The precleared supernatant was then incubated overnight at 4 °C with 10 μg anti-LRP-1 rabbit monoclonal primary antibody (Epitomics, Burlingame, CA, USA), and the antigen-antibody complexes immunoprecipitated by incubating with 50 μl washed protein A/G beads for 1 hour at 4 °C. The beads were then washed 5 times in IP buffer, and the antigen-antibody complex eluted by adding 25 μl buffer for SDS-PAGE (1 × LDS, 1 × dTT, Invitrogen, Grand Island, NY, USA) and heating at 70 °C for 10 minutes.

### Immunoblot analysis

For analysis of 3-nitrotyrosine (3-NT) and HNE modified LRP-1, 5 μl of immunoprecipitated microvascular or parenchymal LRP-1 from saline or LPS-treated mice was resolved in duplicate on a 4–12% Bis-Tris gel (Invitrogen, Grand Island, NY, USA). Protein was then transferred to nitrocellulose membranes using an iBlot transfer device (Invitrogen, Grand Island, NY, USA), washed 5 minutes in PBS-T, and blocked for 1 hour in 5% milk dissolved in PBS-T. All antibody incubations were done for 1 hour at room temperature, except for Pgp which was done at 4 °C overnight. One membrane was probed with anti-HNE mouse monoclonal antibody (R and D systems, Minneapolis, MN, USA; 2 μg/ml) and the other membrane probed with anti-3-NT mouse monoclonal antibody (Millipore, St. Charles, MO, USA; 2 μg/ml). Both membranes were then washed, and probed with anti-mouse secondary antibody conjugated to horseradish peroxidase (Santa Cruz Biotechnology, Inc., Santa Cruz, CA, USA; 1:5,000). Following band visualization, both blots were stripped and confirmed for absence of signal. Blots were then re-probed with an anti-LRP-1 antibody which recognizes the small subunit (Epitomics Burlingame, CA, USA; 0.2 μg/ml), washed, and probed with anti-rabbit secondary (Santa Cruz Biotechnology, Inc., Santa Cruz, CA, USA; 1:10,000). Band intensities for HNE and 3-NT were then normalized for LRP-1 signal. For analysis of LRP-1 in isolated microvessels, 15 μg were loaded on a 3–8% Tris-acetate gel (Invitrogen, Grand Island, NY, USA), and transferred onto a nitrocellulose membrane. Prior to probing for LRP-1, the blot was stained with SYPRO ruby (Invitrogen, Grand Island, NY, USA), and protein bands were quantified under UV exposure for normalization, as described previously. The blot was then blocked, and probed with anti-LRP-1 antibody which recognizes the large subunit (2 μg/ml 1 hour at room temperature), followed by probing with secondary anti-rabbit (1:5,000). The same blot was then re-probed for the small subunit of LRP-1 using the antibody from Epitomics (0.1 μg/ml), followed by anti-rabbit secondary. Immunoblotting of HBEC lysates was done using the same method for isolated brain microvessels, with the following modifications: 8 μg was used for loading, blots were only probed for the small subunit of LRP-1, and bands were normalized to gamma-tubulin (Santa Cruz Biotechnology, Inc., Santa Cruz, CA, USA; 1 μg/ml). Immunodetection of Pgp was done in microvascular extracts by loading 3 μg of protein on a 4–12% Bis-Tris gel under nonreducing conditions. The lower half of the blot was stained with SYPRO ruby, and the upper half probed for Pgp (primary: C219, Covance, Princeton, NJ, USA; 1 μg/ml in 2% milk, secondary: Santa Cruz; 1:5,000). As a significant hook effect has been reported for Pgp [47], it was confirmed that our protein loading conditions fell within linear range of antibody signal for Pgp. All immunoreactive bands were visualized using West Pico chemiluminescent substrate (Thermo Scientific, Rockford, IL, USA) and all images were captured using an ImageQuant LAS4000 CCD imaging system (GE Life Sciences, Piscataway, NJ, USA) except for HBEC blots, which were captured on film. Densitometric analysis was done using IQTL software (GE Life Sciences, Piscataway, NJ, USA).

### Dot blot analysis

RBE4 lysates were diluted to a final concentration of 4 μg/ml in PBS, and 1 μg of protein was loaded onto a nitrocellulose membrane in duplicate using a Bio-dot apparatus (BioRad, Hercules, CA, USA). Membranes were then probed for the small subunit of LRP-1 and analyzed by densitometry as described above. The antibody used for detection is specific for LRP-1, and shows no signal in the PEA-13 knockout cell line [47].

### Immunocytochemistry

MBECs grown on Transwell inserts were washed in PBS and fixed with 4% PFA for 10 minutes at 4 °C. Cells were permeabilized with 0.1% TRITON-X100, blocked with 5% BSA and then incubated with anti-LRP1 rabbit monoclonal antibody (Epitomics, Burlingame, CA, USA) and anti-ZO-1 rat monoclonal antibody (Millipore, St. Charles, MO, USA) followed by incubation with corresponding Alexa Flour-488 or Alexa Flour-568 conjugated secondary antibody (Invitrogen, Grand Island, NY, USA ). Inserts were mounted in antifade media containing DAPI (nuclear) counterstain and photographed with a Nikon ECLIPSE E800 fluorescence microscope.

### Statistical analysis

All statistical analysis was done using Prism 5 software (GraphPad Inc, San Diego, CA, USA). Data from liver and kidney uptake as well as serum clearance were analyzed by linear regression, and the remaining data were analyzed by two-tailed Student’s *t*-tests. Data are shown as mean +/− SEM. In all figures, * *P* <0.05, ** *P* <0.01, and ***P < 0.001 compared to saline.

## Results

### Effects of LPS on vascular sequestration of Aβ and a2M

**Figure 1:**
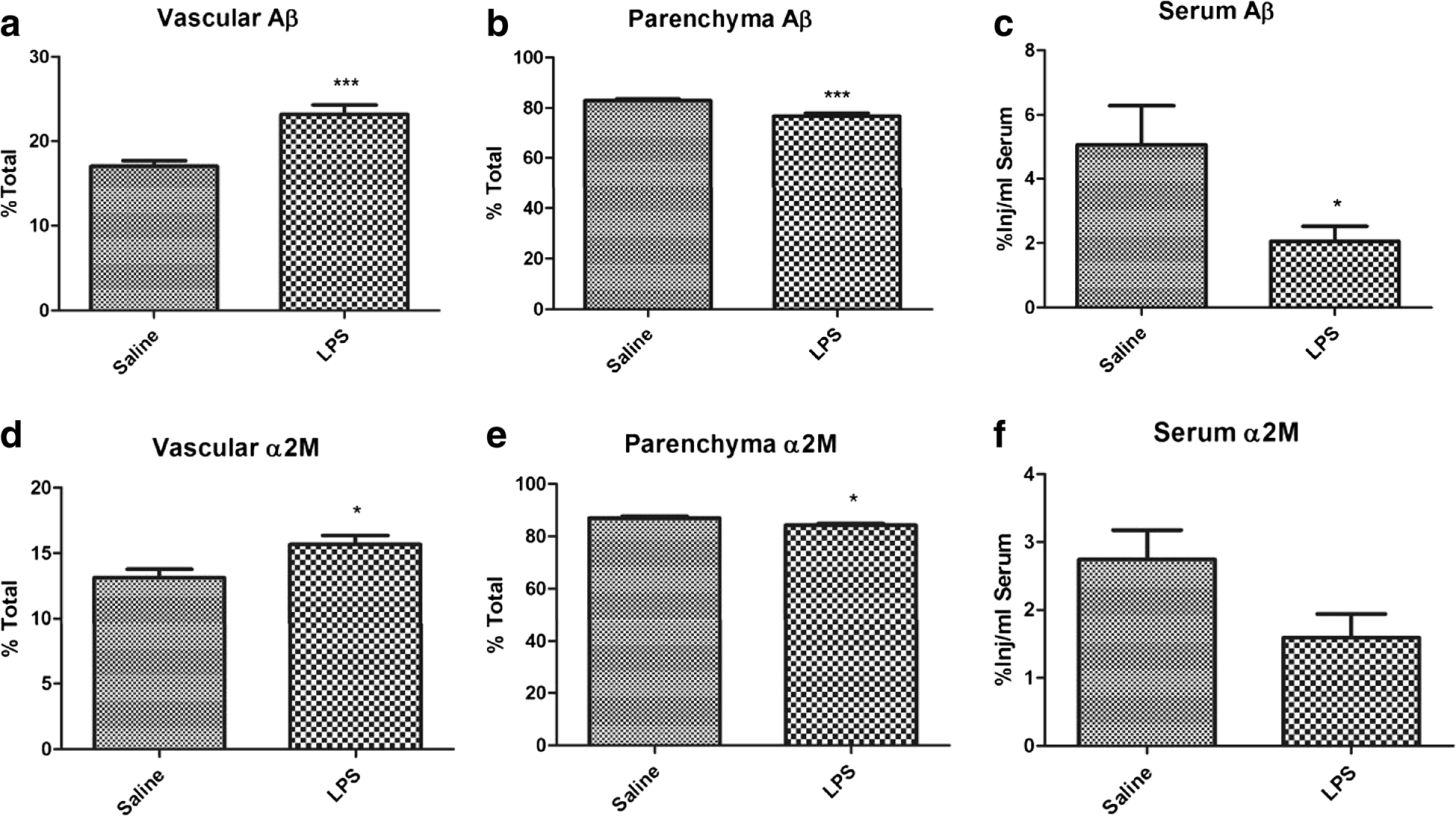
**Distribution of ICV-injected murine**^**125**^**I-Aβ**_**1-42**_**(a–c) or activated **^**125**^**I-a2M (d,–f) in the vasculature (a,d), parenchyma (b,e), and serum (c,f) after treatment with LPS.** Data analyzed by two-tailed *t*-test, *n* = 10–11 per group, *P < 0.05; ***P < 0.001.

To characterize the defect in Aβ transport by brain vasculature, the method of capillary depletion was applied which is routinely used to determine whether circulating compounds are sequestered by brain endothelial cells [42]. In this case, however, it was used to determine whether ICV-injected I-Aβ showed significant changes in partitioning between the brain capillary and parenchymal compartments. We reasoned that decreased vascular partitioning of Aβ would indicate decreased binding/internalization at the abluminal surface, whereas increased vascular partitioning would indicate a post-internalization defect, that is, in the intracellular transport and luminal efflux phases. To establish whether our results reflected an LRP-1-dependent process, the same study was repeated with the LRP-1 ligand a2M. Figure 1 shows that LPS treatment significantly shifted brain distribution of I-Aβ and I-a2M from the parenchymal compartment to the vascular compartment (Figure 1a,b; Figure 1d,e), consistent with an LRP-1-dependent post-binding inhibition of Aβ efflux. To confirm that this was associated with impaired Aβ efflux, venous blood was also collected from these mice just prior to decapitation. I-Aβ in serum was significantly decreased with LPS and I-a2M showed a decreased trend (p = 0.0538; Figure 1c and f), supporting our previous findings that Aβ efflux from brain is inhibited by systemic inflammation [32].

### LPS effects on clearance of Aβ in the periphery

**Figure 2:**
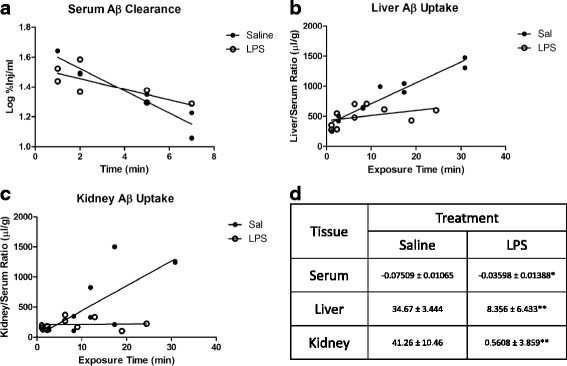
**Peripheral clearance of murine**^**125**^**I-Aβ**_**1-42 **_**from blood (a), and uptake by liver (b) or kidney (c) following LPS.** Clearance rate (%Inj/ml-min) of Aβ from serum and unidirectional influx rates for liver and kidney (μl/g-min) are shown in (**d**). Data analyzed by linear regression analysis, *n* = 7–10 per group, *P < 0.05, **P < 0.01.

Previous studies have shown that Aβ in the circulation is cleared primarily by the liver and less so by the kidneys [16]. Because alterations in peripheral clearance would affect the serum levels of I-Aβ shown in Figure 1, we determined the effect of LPS on Aβ clearance from serum by liver and kidney. Figure 2a shows that clearance of I-Aβ from serum is reduced with LPS treatment (half-life increased from 4 minutes for saline to 8.4 minutes for LPS), and this is coupled with a significantly decreased unidirectional influx rate of Aβ into liver (Figure 2b, Ki = 34.67 ± 3.444 and 8.356 ± 6.433 μl/g-min saline and LPS, respectively) and kidney (Figure 2c, Ki = 41.26 ± 10.46 and 0.5608 ± 3.859 μl/g-min saline and LPS, respectively). Therefore, serum Aβ levels shown in Figure 1c underestimate the magnitude of efflux impairment by LPS.

### Effects of LPS on CSF bulk flow

**Figure 3:**
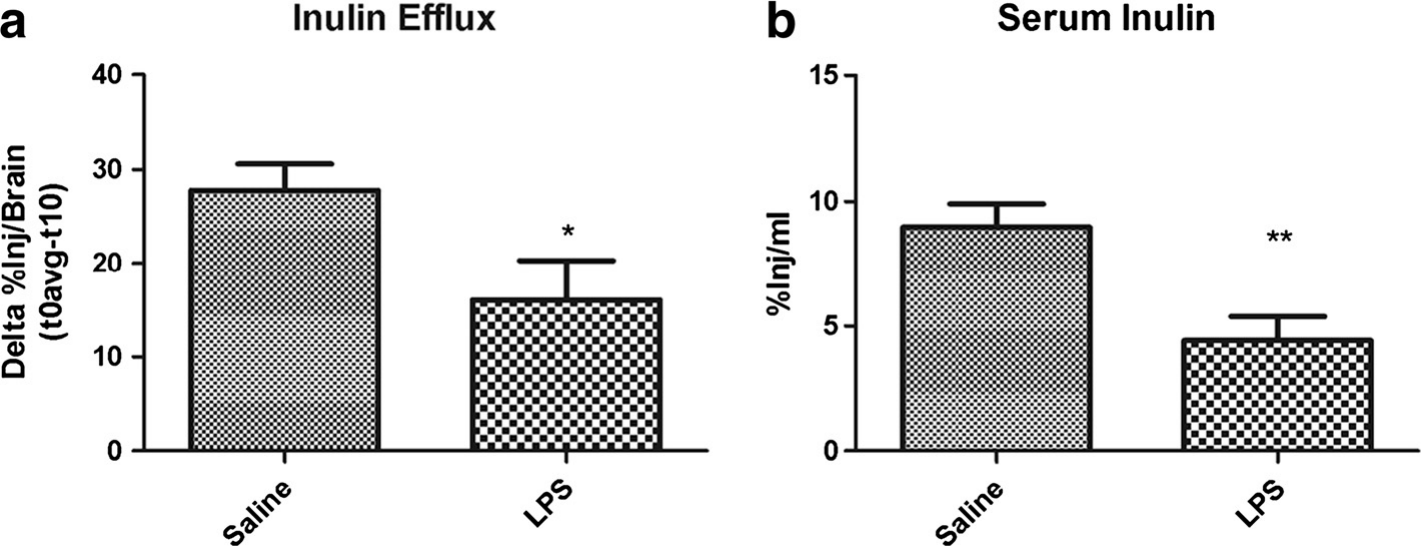
**Brain efflux of ICV-injected**^**14**^ **C inulin (a) and corresponding appearance in serum (b) after treatment with LPS.** Lower values indicate slower efflux. Data analyzed by two-tailed *t*-test, *n* = 10 per group, *P < 0.05, **P < 0.01.

In addition to saturable efflux across the BBB, Aβ clearance from brain through bulk flow of CSF partially contributes to our measures of total Aβ efflux. To test the effects of LPS on CSF bulk flow, we measured brain efflux of the bulk flow marker inulin [4]. Figure 3a shows disappearance of ^14^ C-inulin from brain after 10 minutes, corrected for its CNS distribution at time zero. Therefore, the 42% decrease in the delta value indicates that LPS treatment significantly decreases CSF bulk flow. Figure 3b shows that serum levels of ^14^ C-inulin also significantly decrease with LPS, further demonstrating that CSF bulk flow is impaired in this model.

### Brain microvascular expression of LRP-1 and pgp, and oxidative modification of LRP-1

**Figure 4:**
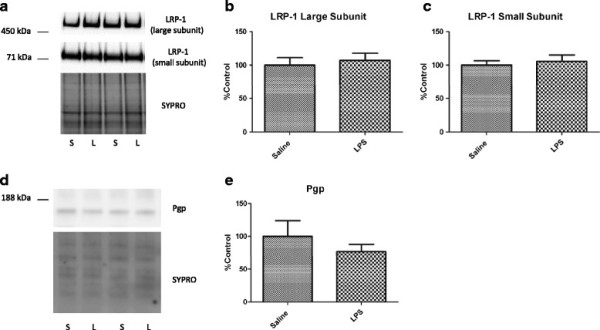
**Protein expression of LRP-1 and Pgp in pooled brain microvessels isolated from mice treated with saline or LPS.** Representative immunoblot images shown for LRP-1 (**a**) and Pgp (**d**). Results from densitometric analysis of the small (**b**) and large **(c)** subunits of LRP-1, and Pgp (**e**) are shown as bar graphs. Data analyzed by two-tailed *t*-test, *n* = 4–5 microvessel pools (3 brains/pool) per group.

**Figure 5:**
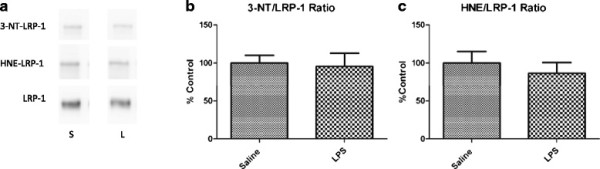
**Oxidative modifications to brain microvascular LRP-1 following LPS treatment.** Representative immunoblots for 3-nitrotyrosine (3-NT) and 4-hydroxynonenal (HNE)-modified LRP-1 and immunoprecipitated LRP-1 are shown in (**a**). Results from densitometric analysis of 3-NT (**b**) and HNE (**c**)-modified LRP-1 normalized to total LRP-1 levels are shown as bar graphs. Data analyzed by two-tailed *t*-test, *n* = 4–5 microvessel pools (10 brains/pool) per group.

To determine effects of LPS on efflux transporter expression, we first measured levels of LRP-1 and Pgp in brain microvessels isolated from mice treated with LPS or saline. Figure 4 shows that neither LRP-1 (Figure 4a–c) nor Pgp (Figure 4d,e) levels are altered significantly with LPS in isolated brain microvessels. Because it was found that oxidative modification of LRP-1 significantly increases in the AD hippocampus, and inflammation is associated with increased oxidative stress in the brain, we determined whether similar patterns in oxidative modification were present in isolated brain microvessels in our model. Figure 5 shows that no significant alterations in oxidative modification of LRP-1 occur with LPS. Interestingly, oxidative modifications to LRP-1 were not detectable in capillary-depleted brain homogenate from either group tested (data not shown).

### LPS effects on LRP-1 expression *in vitro*

**Figure 6:**
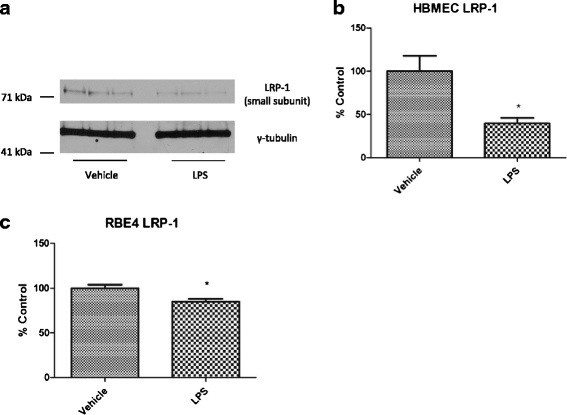
**Decreased protein expression of LRP-1 in cultured HBECs and RBE4 cells following LPS treatment.** Immunoblots of LRP-1 and γ-tubulin from HBECs are shown in (**a**), and densitometric analysis of LRP-1 small subunit expression shown in (**b**). Data from dot blot analysis of LRP-1 in RBE4 cells is shown in (**c**). Data analyzed by two-tailed *t*-test, *n* = 3 per group (HBEC) or 6 per group (RBE4), *P < 0.05.

**Figure 7:**
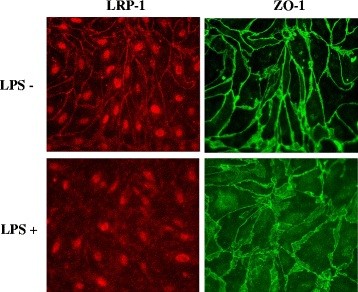
**Mislocalization of LRP-1 and ZO-1 in cultured MBECs following LPS treatment.** MBECs grown on Transwell inserts were treated with LPS and stained for LRP-1 (red), ZO-1 (green), and DAPI (blue). Images captured at 400× magnification.

Although no changes were found for LRP-1 expression in isolated brain microvessels following LPS treatment *in vivo*, we have recently found that cultured brain microvascular pericytes upregulate LRP-1 when treated with LPS *in vitro*[48]. Mechanical preparations of isolated brain microvessels include pericytes, due to their juxtaposition to endothelial cells [49]. This raises the possibility that pericyte upregulation of LRP-1 masks downregulation of LRP-1 at the endothelial cell. To test this, we treated primary cultures of HBECs, the rat brain endothelial cell line RBE4, and primary cultures of mouse brain endothelial cells (MBECs) with LPS and measured LRP-1 protein expression or localization following treatment. Figure 6 shows that LRP-1 is significantly downregulated following LPS treatment in both HBECs (Figure 6a,b) and the RBE4 cell line (Figure 6c). We were unable to detect Pgp in primary pericytes using the same antibody (data not shown). In addition to downregulation, Figure 7 shows that LRP-1 mislocalization occurs following LPS treatment in MBECs. This change is associated with mislocalization of the tight junction protein ZO-1.

## Discussion and conclusions

**Figure 8:**
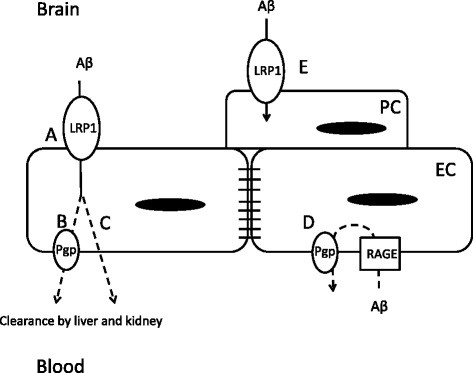
**Schematic of mechanisms at the neurovascular unit which contribute to LPS-induced decreases in Aβ efflux by the BBB.** Decreased efflux at the brain endothelial cell (EC) is due to functional impairment of LRP-1 (**a**) that may involve (**b**) or not involve (**c**) Pgp (**b**). Impairment of Pgp could also result in increased vascular uptake of Aβ from the periphery (**d**). Increased internalization of Aβ in the pericyte (PC) through upregulation of LRP-1 may also contribute to Aβ partitioning in the brain vasculature (**e**). Dashed line across endothelial cell indicates unknown subcellular routes in the translocation of Aβ.

In this study we have shown that peripheral administration of LPS inhibits CSF bulk flow, central and peripheral clearance of Aβ, and increases vascular sequestration of Aβ. All four of these positive results demonstrate that systemic inflammation alters the distribution of Aβ in ways that would favor its accumulation in brain. Other groups have shown LRP-1-dependent efflux of human forms of Aβ across the BBB [4,50], however, we have observed that murine Aβ_1-42_ also undergoes LRP-1-dependent BBB transport and is cleared at a faster rate across the murine BBB than human Aβ_1-42_[7,51]. Although we did not observe direct changes to LRP-1 or Pgp *in vivo*, our findings of increased vascular partitioning of Aβ and decreased LRP-1 expression in cultured brain endothelial cells provides important clues of how BBB transport dysfunction could occur, as modeled in Figure 8.

The finding that inulin efflux from brain is decreased following LPS administration demonstrates that CSF/interstitial fluid (ISF) bulk flow decreases in our model. CSF/ISF turnover is important for clearing catabolites from the brain, and maintaining an optimal environment for neuronal function [14,52]. It has been shown that substantial catabolite buildup occurs after a 50% decrease in CNS fluid turnover rate, and this can lead to neuronal toxicity [14]. This rate of decrease is observed in AD, and therefore may contribute to toxic catabolite buildup in the AD brain [52]. In healthy animals and humans, the magnitude of Aβ efflux by the saturable BBB systems is much greater than clearance by bulk flow [4], but transporter deficiency could result in a shift where bulk flow becomes the predominant clearance route. In our LPS model we observed a 42% decrease in inulin efflux compared with control. This shows that similar to AD, bulk flow is also impaired during systemic inflammation. Future studies are necessary to determine to what extent this deficit contributes to cognitive dysfunction.

In addition to its contribution to bulk flow, the blood–CSF barrier is likely to play important roles in Aβ removal from CSF. Epithelial cells of the choroid plexus have saturable transport systems for Aβ [53], and LRP-1 at the choroid plexus participates in the clearance of Aβ from the CSF [54]. This may have important implications for AD because LRP-1 expression increases in the rat choroid plexus with age [55]. ICV-injected Aβ would be subject to transport by LRP-1 at the BBB and blood–CSF barrier, and the relative contribution of each route to total clearance is presently unclear. Whether LPS alters LRP-1 function at the choroid plexus is also unknown, but this could reflect an additional mechanism involved in the observed Aβ clearance deficit.

Peripheral clearance of Aβ from the circulation is primarily dependent on uptake by the liver and kidneys [16]. In this study we measured clearance of Aβ from blood along with liver and kidney uptake to confirm that brain-derived Aβ contributes substantially to measurements of Aβ in blood. The finding that Aβ levels were decreased in blood following LPS even though a decrease in peripheral clearance was observed shows that serum levels of exogenous Aβ underestimate the magnitude of LPS inhibition on Aβ efflux from brain. Despite this, we found that LPS causes a significant reduction of ICV-injected Aβ in serum, which supports previous results that LPS impairs Aβ efflux from brain. The observation of decreased Aβ clearance from blood by both liver and kidney is also of interest. Because LRP-1 mediates peripheral clearance of Aβ in liver [17] our findings suggest LRP-1 impairment may be occurring with LPS at peripheral sites. Whether Aβ clearance by kidney is also mediated by LRP-1 is unknown, but reflects a likely route as LRP-1 is expressed in kidney. LRP-1 is known to be cleaved in response to inflammatory stimuli [33], which may reflect a mechanism for peripheral LRP-1 dysfunction in our LPS model. Reduced peripheral clearance of Aβ may be contributing to Aβ accumulation in brain through increased BBB influx via RAGE [18,32], as well as by decreasing Aβ efflux [19]. The latter mechanism may be explained either by decreases in the Aβ concentration gradient between brain and blood, or endothelial dysfunction through RAGE activation. Because studies indicate that processes regulating circulating Aβ play important roles in AD pathogenesis [56,57], these results highlight a novel mechanism by which systemic inflammation could contribute to AD.

Our findings of decreased brain efflux and increased neurovascular Aβ sequestration with LPS could be explained by the interaction of several pathways as illustrated by the working model in Figure 8. The first consideration of Figure 8 is the subcellular location of the efflux transporters LRP-1 and Pgp. The model presented here, in line with a recently reported model [11], starts with extracellular Aβ first coming into contact with LRP-1 on the abluminal side of the brain endothelial cell (Figure 8a). This is followed by transport of Aβ into the vascular lumen by Pgp (Figure 8b), or by a Pgp-independent pathway (Figure 8c). Entry of circulating Aβ into the brain is mediated by RAGE, but can also be restricted by Pgp [39] (Figure 8d). Our result showing that vascular Aβ and a2M partitioning increases with LPS is consistent with a functional deficit in LRP-1. This would not, however, be due to mechanisms that decrease binding interactions between Aβ and LRP-1 such as ligand competition [58,59] because this would be expected to decrease vascular partitioning. Along these lines, our observations of decreased expression of LRP-1 at the endothelial cell are inconsistent with increased vascular partitioning unless other cells of the neurovascular unit are considered. LRP-1 expressed in other cells tightly associated with the neurovasculature such as pericytes and vascular smooth muscle cells could contribute to vascular partitioning [60,61] because these cells remain associated with the vascular pellet. Therefore, increased vascular partitioning may also reflect an increase in uptake of Aβ by pericytes or vascular smooth muscle cells (Figure 8e). Interpretation of the results for vascular sequestration is discussed below in context of our findings for transporter expression and what is known from the literature about LRP-1 and Pgp regulation.

Our *in vivo* findings for BBB transporter expression showed no significant changes in LRP-1 or Pgp expression following LPS treatment, nor did we find increases in oxidative modifications to LRP-1. Interestingly, we did find that oxidative modifications to LRP-1 which were detectable in brain endothelial cells of saline-treated animals were not detected in capillary-depleted parenchymal fractions from the same isolation, despite strong immunostaining for immunoprecipitated LRP-1 (data not shown). Although LRP-1 is generally considered to be a receptor that recycles from membrane to lysosomal compartments, it has been shown that phosphorylation can regulate this process [62]. Oxidative modification to LRP-1 could alter such processes, impairing unique physiological functions, such as transcytosis at the BBB. Recently, it has been shown that LRP-1 transcytosis occurs in an *in vitro* BBB model [63]. Future studies are necessary to determine whether post-translational modifications are needed to confer unique functions to LRP-1 at the BBB.

Despite our inability to show LPS-induced changes in BBB LRP-1 *in vivo*, we did find that LRP-1 expression was significantly decreased by treating cultured HMECs or RBE4 cells with LPS *in vitro*. This is opposite to the effect we recently reported for primary cultured pericytes, which upregulate LRP-1 in response to LPS [48]. Therefore, the possibility is raised that upregulation of LRP-1 at the pericyte masks downregulation of LRP-1 at the endothelial cell, which would explain our findings of decreased BBB efflux in the absence of changes in protein expression *in vivo*. Furthermore, increased expression of LRP-1 at the pericyte could also explain our finding that the distribution of ICV-injected Aβ shifts from the parenchyma to the vascular compartment. Mislocalization of LRP-1 was also found to occur with LPS in primary brain endothelial cells, further supporting that LPS induces LRP-1 dysfunction at the brain endothelial cell. Because LRP-1 participates in Aβ internalization and transport *in vitro*[50,60,63], future studies are necessary to determine how these *in vitro* changes in LRP-1 alter the cellular uptake and transport of Aβ.

It is possible that in addition to LRP-1, Pgp dysfunction may also contribute to increased vascular partitioning and decreased BBB efflux of Aβ. Although we found no changes in Pgp expression *in vivo* following LPS, this does not contradict other reports in the literature where Pgp is functionally inhibited despite unchanged or upregulated protein expression [36,37,64,65]. Furthermore, we and others have found that the LPS regimen used in this study impairs Pgp function at the BBB [38,64]. Therefore, Pgp dysfunction could also be mediating defects in Aβ transport. Our inability to show a significant decrease in Pgp expression also signifies an important difference between our model and AD because Pgp is downregulated in the brain microvasculature in AD [12].

Together, these results show that BBB Aβ transport is functionally impaired following an inflammatory response. Although our *in vitro* data suggest that LPS downregulates LRP-1 at the brain endothelial cell *in vivo*, the lack of any significant decrease in Pgp expression highlights an important distinction from AD, where both LRP-1 and Pgp are downregulated in the brain microvasculature. An explanation for this distinction may be related to our use of young adult mice in this study. Because Aβ is produced under normal physiological conditions, and our data utilizes an acute model of systemic inflammation [66], it may be that the mechanistic differences observed for Aβ efflux deficiency in inflammation and AD represent components of a physiological process. Aβ has been implicated as a stress-response molecule [67], and at physiological levels has neuroprotective properties [30,68]. Hence, it is tempting to speculate that downregulation of BBB efflux transporters in AD may represent a pathological consequence of prolonged vascular sequestration of Aβ as a result of sustained systemic inflammation. This possibility is supported by another group who showed in a transgenic model of AD that Pgp dysfunction at the BBB precedes symptoms of cognitive impairment, and that microvascular upregulation of LRP-1 also occurs at this time point [11]. Furthermore, cerebrovascular accumulation of Aβ is cytotoxic [61]. Because low-grade systemic inflammation is associated with many other diseases which have been considered comorbidities in AD [69], similar comparative studies would be useful in determining unifying pathological events at the neurovascular unit or related to brain fluid dynamics which would contribute to impaired Aβ clearance from the brain. Aging would likely sensitize an organism to inflammation so that the threshold required for Aβ efflux impairment is lowered [70]. In conclusion, we have shown that inflammatory events at the neurovascular unit affect key players that regulate the brain and blood levels of Aβ, providing mechanistic pathways by which inflammation could promote or even induce important characteristics of AD.

## Competing interests

The authors declare that they have no competing interests.

## Authors’ contributions

ME participated in the conception and design of the study, carried out or oversaw all experimental studies and drafted the manuscript. PEH carried out the measurements of systemic clearance of Aβ, and assisted in drafting the manuscript. YM carried out the primary endothelial cell and RBE4 cultures and treatments, as well as the immunofluorescence measurements of LRP-1 and ZO-1, and assisted in drafting the manuscript. JBO assisted with tissue processing, provided technical assistance for the measurement of LRP-1 oxidation, and assisted in drafting the manuscript. DAB assisted with the conception and design of the study. WAB conceived and oversaw the design and coordination of the study, and assisted in drafting the manuscript. All authors read and approved the final manuscript.
